# Transcending Self Therapy Virtual Reality Expansion: Effect on Engagement, Completion and Temporal Usage Patterns

**DOI:** 10.1177/29941520251372153

**Published:** 2025-09-12

**Authors:** Anna M. Wiese, Jarrod Reisweber, Mark Lambert, Ginna Lambert, Joseph Gauntlett, James M. Bjork

**Affiliations:** ^1^ Department of Psychology, Virginia Commonwealth University, Richmond, Virginia, USA.; ^2^ Central Virginia VA Health Care System, Richmond, Virginia, USA.; ^3^ Lighthouse XR, Richmond, Virginia, USA.; ^4^ VA Mid-Atlantic Mental Illness Research, Education, and Clinical Center, Durham, North Carolina, USA.

**Keywords:** substance use disorder, Transcending Self Therapy (TST), veterans, virtual reality, CBT

## Abstract

Transcending Self Therapy (TST) is an integrative cognitive behavioral therapy for substance use disorders (SUDs) that was adapted into an immersive virtual reality (VR) platform (TST-VR) featuring psychoeducation modules, mindfulness exercises, and interactive activities that reinforce TST principles. Utilizing an evidence-informed framework and incorporating feedback from a feasibility study, Transcending Self Therapy Virtual Reality (TST-VR) was expanded from a 4-Module version to a 10-Module version. Each of these modules contains four to six distinct lessons or interactive activities presented in a virtual environment. The aim of this study was to examine the effect of this expanded content on engagement with TST-VR. We hypothesized that the expanded content and feedback-informed changes incorporated into the 10-Module version would increase engagement in terms of total time (minutes) in the program and program completion. Fifty-eight military veterans receiving or being considered for SUD treatment (*N* = 34 in the 4-Module group; *N* = 24 in the 10-Module group) received access to TST-VR headsets. Usage metrics, including total time in headset, time of day usage, and module completion, were tracked to assess adherence and patterns of engagement. Contrary to concerns that more modules could increase attrition, veterans assigned the 10-Module program not only spent a higher average total time in headset but also showed a greater likelihood of completing all assigned modules. In an exploratory analysis, temporal analyses further revealed that overnight usage (midnight to 6 AM) was observed in a sizable minority in both groups (14% of users in 4-Module, 29% of users in 10-Module), suggesting that VR can function as an on-demand therapeutic resource beyond typical clinical hours. Additional insights were indicated, such as the counterintuitive finding that, in a population that may not be digitally literate with gaming devices, the amount of time spent in the early tutorial section (learning how to use the device controls) did not indicate whether a user would complete all Modules. These data imply that following user-centered design principles and expanding VR content (creating a larger, more fulfilling world) not only sustains but may increase patient engagement, warranting further study into how a larger virtual world can foster adherence and SUD treatment engagement.

## Introduction 

Substance use disorders (SUDs) among veterans constitute a costly and complex public health challenge, compounded by high rates of comorbidities like post-traumatic stress disorder (PTSD) and traumatic brain injury (TBI).^1,2^ Psychotherapy such as the cognitive behavioral therapy (CBT)-based Transcending Self Therapy (TST) has shown promise in improving SUD treatment outcomes at VA Medical Centers.^3^ TST is a form of CBT. In addition to helping patients change maladaptive thoughts and behaviors, TST contends that it is important for patients to identify their moral compass and how to live life in accordance with their morals. Patients being treated with TST are encouraged to understand the thinking and behavior patterns that interfere with a value-oriented life as well as what they need to do to develop healthy interpersonal relationships and a passionate pursuit/meaningful life. The success of TST in its group and individual talk therapy formats led to an interest in investigating other avenues for delivering TST to patients.

**FIG. 1.**
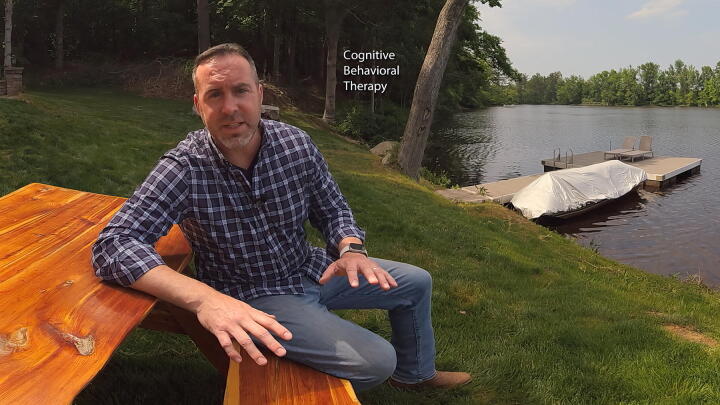
Image from the Transcending Self Therapy Virtual Reality (TST-VR) CBT Modules.

To this end, our previous work examined the feasibility of expanding TST into an immersive virtual reality (VR) environment Transcending Self Therapy Virtual Reality (TST-VR) to provide self-guided therapeutic engagement and immersive psychoeducation for veterans undergoing residential SUD treatment (Fig. 1).^4^ We did so by following a clinical VR development framework (the VR-CORE Model^5^) that has three phases: VR1, VR2, and VR3. In this framework, VR1 studies focus on content development through human-centered design with patients and providers, VR2 trials conduct feasibility/acceptability/tolerability testing and initial clinical efficacy, and VR3 trials are randomized controlled studies or demonstration projects evaluating efficacy.

Applying this framework, our initial feasibility study of a single Module prototype (VR1)^4^ obtained feedback that informed initial design and iterative refinements to TST-VR, wherein pilot participants reported positive reactions to the virtual environment. Based on this promising initial feedback, the program was expanded from one to four Modules of psychoeducation and activity content, and a VR2 study was undertaken. During this VR2 study, the program was further expanded from 4 Modules to 10 Modules. The initial 4-Module version focused mainly on core CBT concepts such as catching, checking and changing thoughts. For the 10-Module version, several existing videos were rewritten and reshot based on feedback, and new Modules were added around the following topics: New Beginnings, The Moral Compass, Relapse and Chain Analysis, Anxiety, Anger, and Behavioral Activation. The 10-Module version also incorporated ongoing user and clinician feedback to allow for smoother interactive gameplay. Changes were also added to support all levels of users with refined instructions and improved and more detailed feedback if the program detected that a user misunderstood instructions.

We solicited anonymous patient feedback for both versions, which indicated that the program was engaging and helpful to recovery efforts, with most respondents reporting that TST-VR was important to their recovery and providers reporting no systematic age-related technical barriers.^4^ A subsequent program evaluation based on the 10-Module version also found that veterans who opted to participate in the VR program were significantly more likely to complete treatment than those who did not.^6^

**FIG. 2.**
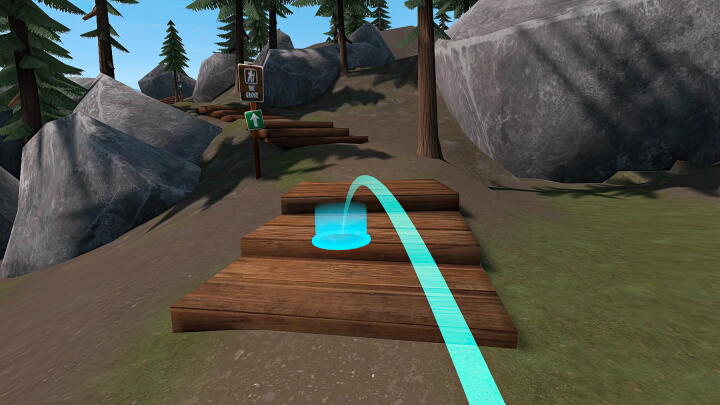
Image of navigating in The Retreat platform virtual world.

Evidence suggests a significant positive relationship between increased homework compliance, longer treatment duration, and improved substance use disorder treatment outcomes.^7,8^ However, with mobile-based digital treatments, multiple studies have pointed to high attrition rates and poor rates of sustained engagement as a potential pitfall to using digital applications.^9^ We expected the addition of more content and user-centered design changes would lead to better engagement, but a key concern is whether adding more Modules or activities might increase the risk of dropout due to “digital fatigue” or overwhelm the user. There was potential for the 10-Module expansion to reduce engagement, as a meta-analysis of treatment outcomes found that for in-person SUD treatment, more sessions were associated with higher dropout rates.^10^ Relatedly, patients could be turned off once they discover the prospect of having to complete more Modules, considering deficits in motivation toward non-drug rewards are common in SUD.^11^ Patients’ willingness to consider engaging in the larger virtual world could also be affected by the “paralysis by analysis” evidenced in consumer choice studies, wherein presentation of additional choice options beyond a handful actually reduces engagement (purchases).^12^ Conversely, there was also the potential for expanded content to increase overall engagement in the virtual therapeutic world based on the experiences of virtual world video game platforms, where periodic expansions of virtual world content frequently elicit greater engagement or re-engagement with the virtual world. If those phenomena were to extend to a therapeutic virtual world, participants may show a higher completion rate when there is expanded VR content (a larger, more fulfilling experience) in a new world that is distinct from their own and enjoyable to navigate and explore (Fig. 2).

The present study thus explored these issues with a sample of 58 sequentially consented veterans who were in residential SUD care shortly before and after the transition from the 4-Module to the 10-Module TST-VR variant. We examined whether veterans assigned to the 10-Module TST-VR format would maintain or even exceed the engagement and completion rates observed with the 4-Module version. We hypothesized that despite the potential for added content to appear daunting, especially to technologically novice users, the added content of the 10-Module version would nevertheless increase total minutes of program engagement and program completion. As an exploratory analysis, we further sought to capture temporal patterns of use, including the prevalence of overnight or other off-hours engagement. We also assessed time spent to complete an early tutorial section as a potential marker of program completion. Understanding how participants respond to an expanded VR environment has broader implications for VR-based psychoeducation in SUD treatment and may inform future refinements to the TST-VR program.

## Methods

All recruitment and testing procedures were reviewed and approved by the Institutional Review Board of the local Veterans Affairs Medical Center (VAMC).

### Participants

Participants were *N* = 58 adults (mean age 50.0 years; 90.3% male; 9.7% female; Table 1) recruited from a Mid-Atlantic VAMC who were receiving residential care for one or more SUDs. Participants provided written informed consent to participate. Patients were excluded if they presented with a history of legal blindness, epilepsy or other seizures, or insertion of a pacemaker or other magnetism-susceptible medical device. Recruitment occurred from December 2021 to December 2023. Patients were introduced to the TST-VR by trained study staff and provided with a headset if eligible and interested.

**Table 1.** Demographic Characteristics

Characteristic	4-Module TST-VR group *N* = 34 *N* (%)	10-Module TST-VR Group *N* = 24 *N* (%)	Group difference (*t*-test, chi-square *p* value)
Age (mean [SD])	50.0 (11.1)	48.1 (12.7)	0.563
Sex			0.802
Male	33 (97.0)	23 (95.8)	
Female	1 (3.0)	1 (4.2)	
Race			0.594
White	20 (58.8)	12 (50.0)	
Black	14 (41.2)	12 (50.0)	
Ethnicity			0.746
Hispanic	2 (5.9)	1 (4.2)	
Non-Hispanic	29 (85.3)	22 (91.6)	
Ethnicity Not Reported	3 (8.8)	1 (4.2)	

### Procedures

Following collection of consent, study staff issued a sanitized headset to the participant and helped him or her don the VR equipment. The staff person then verbally guided each participant through a tutorial of how to utilize the TST-VR (that took place in the virtual world from the participant’s perspective). Data on time spent in the headset in each of several discrete virtual locations/activities was then collected across the entire period of gear issuance. Usage data were then offloaded to a secure research server after the participant’s residential stay (or participation) concluded.

### TST-VR

TST-VR allows patients to explore a variety of instructional videos, meditations, and psychoeducational interactive activities while in the setting of a virtual nature landscape. The user is presented with a virtual world, a cabin on a lake, which they can navigate. The information is broken into individual “Modules” based around a central topic. Each Module presents the user with a video introduction, then guides the user to four to six conceptually interrelated lessons or activities to complete, and the user is then guided to a review. Bonus material is unlocked at the end of each Module to provide additional experiences related to the topic or other distraction-based content. More details regarding the specific hardware and content of TST-VR can be found in Zaur et al.^4^

### Data analytic plan

The TST-VR headset software utilizes a proprietary method to record data, including temporal data such as dates, time of engagement, and additional data, to a HIPAA-compliant encrypted file (NIST SP 800–111 compliant). Total time spent in the headset, in minutes, was logged for each participant via the TST-VR headset software, as was module completion. Independent samples t-tests were conducted to determine the association between the TST-VR headset group (4-Module vs. 10-Module) as the independent grouping variable and total time spent in the headset (primary analysis) and other usage metrics (exploratory analyses) as dependent variables. A chi-square analysis compared rates of completion of all modules of that veteran’s VR program as a function of VR program (4-Module vs. 10-Module variant). Independent group two-tailed t-tests and one-way analyses of variance were used to compare groups in quantitative metrics (i.e., minutes). For subsequent analyses, “engagement” was calculated as the time interval between when a participant puts on the headset and begins interacting with the program until they log out, turn the headset off without logging out (in which case the last recorded timestamp was used), or take the headset off and it “times out” after 30 s. A single engagement may involve interaction with multiple TST-VR Modules; for instance, a user could complete Module 3 and begin (or complete) Module 4 in a single engagement. Alternatively, a user is not required to finish a Module in a single engagement and may take two or more engagements with the headset to complete the Module.

In addition to total engagement time and module completion, we examined time-of-day usage patterns by extracting engagement timestamps. Timestamps were grouped into hour-of-day bins (00:00–00:59, 01:00–01:59, etc.) to compute the proportion of participants using the device at each segment, the number of unique engagements, and the total time spent within those segments. This allowed us to explore whether usage occurred predominantly during standard clinical hours versus overnight. In addition, we created a bin to collect the amount of time spent in the tutorial section of Module 1 to analyze whether time spent in this section related to patterns of future use. For example, greater time spent in the tutorial could indicate difficulty mastering its virtual motoric (handset-driven) and navigation tasks, to potentially reduce engagement in the main TST-VR content. Alternatively, greater time spent in the tutorial could itself reflect general motivation for VR immersion, to be a prognostic marker of greater TST-VR engagement in the entire program.

## Results

### Module completion time and engagement time

**FIG. 3.**
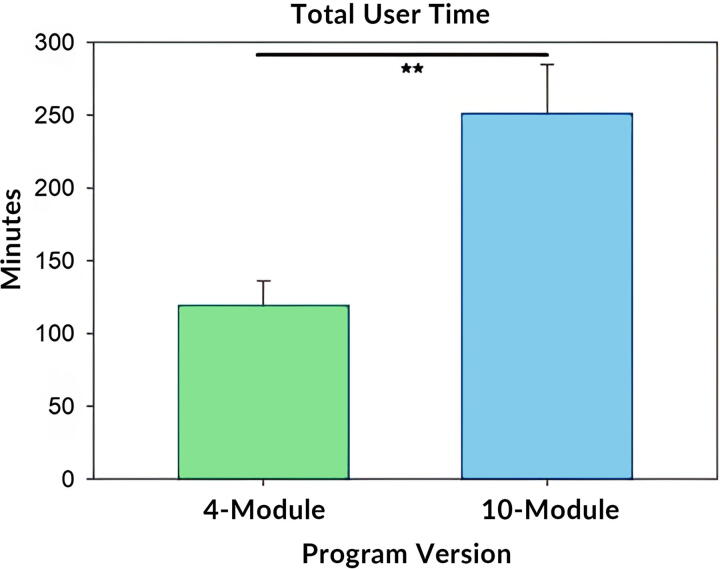
The column graph illustrates mean + SEM values for total VR engagement time across the patient’s entire time span of equipment issuance. Total engagement time varied widely between participants in each program version group. The ** bar denotes difference between groups at *p* < 0.01 per independent *t*-test.

Individuals who participated in the 10-Module version of TST-VR (M = 251.8) spent significantly more time in the VR headset than those who participated in the 4-Module version (M = 120.1; *p* = 0.001; Table 2, Fig. 3).

**Table 2.** TST-VR: Total Time in Headset and Program Completion by Version

	4-Module TST-VR (*N* = 34)	10-Module TST-VR (*N* = 24)	*p* value
Minutes Spent in Headset M (SD) (range)	120.1 (93.6) (22.1–437.0)	251.8 (160.9) (41.0–563.4)	0.001^*^
completed All Sessions *n* (%)	6 (17.7%)	15 (62.5%)	0.0005^**^

^*^ *p* value from independent-samples *t*-test t ≈ 3.60, df ≈ 35).

^**^ *p* value from Pearson chi-square ≈ 12.254, df = 1).

**FIG. 4.**
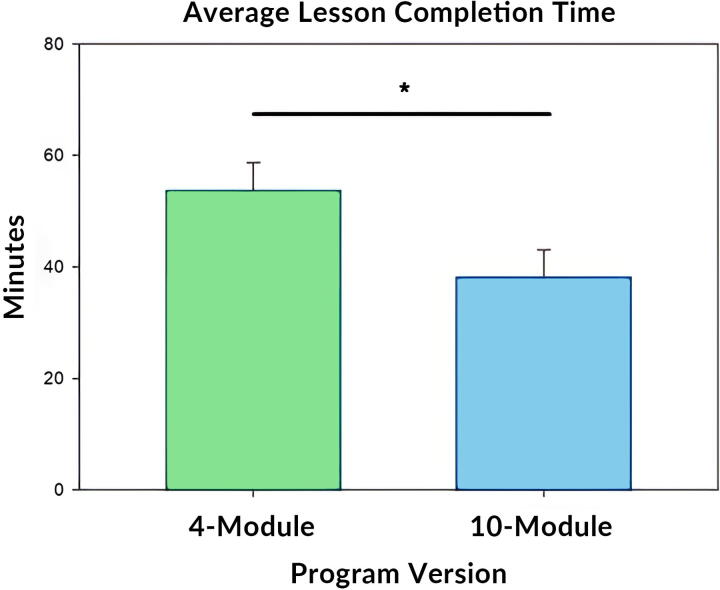
The column graph shows the meantime + SEM a user engaged with each of the distinct TST-VR modules. The * bar denotes difference between program version groups at *p* < 0.05.

We also observed a significantly longer average Module completion time in the 4-Module group compared with the 10-Module group (Fig. 4). This is consistent with design changes in the 10-Module version, most significantly by limiting the maximum amount of content in a single module, and instead distributing some content across more discrete Modules of lower duration. This change was made based on user input about the original 4-Module version. We observed a few outliers (not illustrated) who were users who spent a long time in the headset for 1 or 2 days but did not complete further modules.

**FIG. 5.**
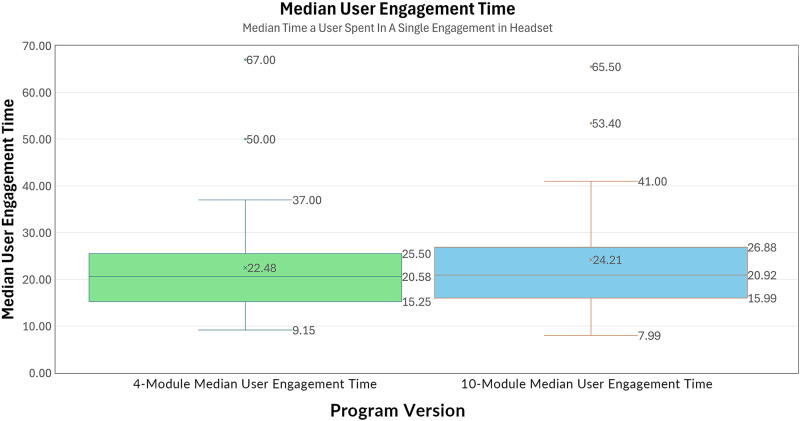
The box and whisker plot shows the 25th, 50th (median), and 75th percentiles of the participants’ median user engagement time in 4-Module and 10-Module program version groups, while also illustrating some outlying cases. The whiskers extend from each box to the furthest data points within 1.5 times the interquartile range (IQR). There was no significant program version group difference in median engagement time per VR usage.

The median times each participant spent in a single engagement (the time spent from when they put on a headset to when they took it off, regardless of whether they finished a module) were also calculated and are shown in Figure 5. There were no significant differences between the two program groups, suggesting that the amount of time required to complete a module or content changes between the two program versions did not affect the amount of time a participant dedicated to using the headset in a single usage. There were high-engagement outliers, but the median of ∼20 min of usage in a single sitting was consistent for both groups.

### Module completion

**FIG. 6.**
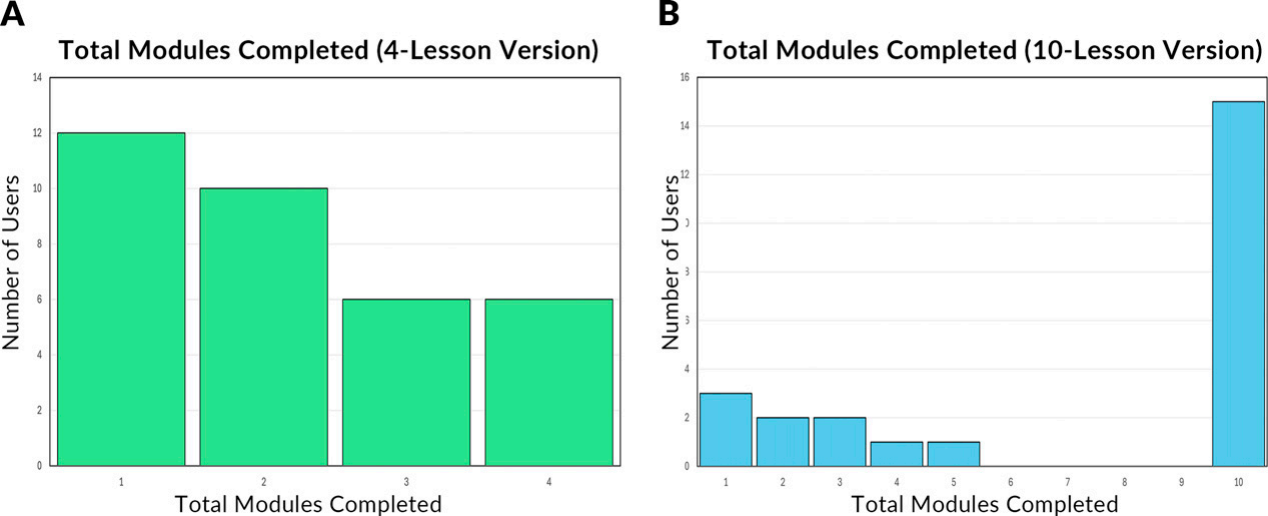
Column tallies illustrate the distribution of individual participant module completion in each of the 4-Module version (Part **A**), and the 10-Module version (Part **B**).

Whereas the majority of participants in the 4-Module version completed only the first two modules (Fig. 6A), a slight majority of participants in the 10-Module version completed all modules (Fig. 6B). A chi-square analysis indicated that the difference between program version groups in incidence of completion of all modules was significant (Table 2).

### Additional temporal usage patterns

**FIG. 7.**
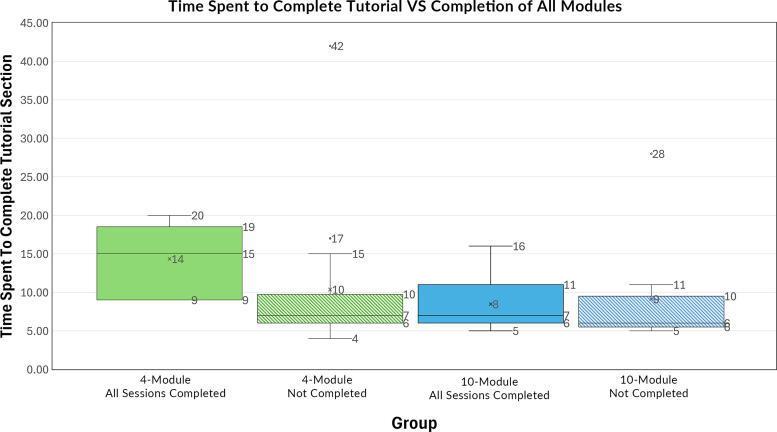
The box and whisker plot shows the 25th, 50th (median), and 75th percentiles of the participants’ engagement time in the instructional tutorial of the 4-Module and 10-Module program version groups, while also illustrating some outlying cases. The whiskers extend from each box to the furthest data point within 1.5 times the interquartile range (IQR). There was no significant program version group difference in tutorial time.

Module 1 includes a brief tutorial to train the user on the digital controls for how to move around the environment and interact with elements in the environment. This tutorial was meant to take around 7 min to complete, though some users who were new to digital/gaming technology took longer to grasp the techniques. In the 4-Module version participants, remarkably, a participant who had to spend more time to complete the initial tutorial corresponded to a greater incidence of completed modules (but this was not seen in 10-Module version participants) (Fig. 7).

**FIG. 8.**
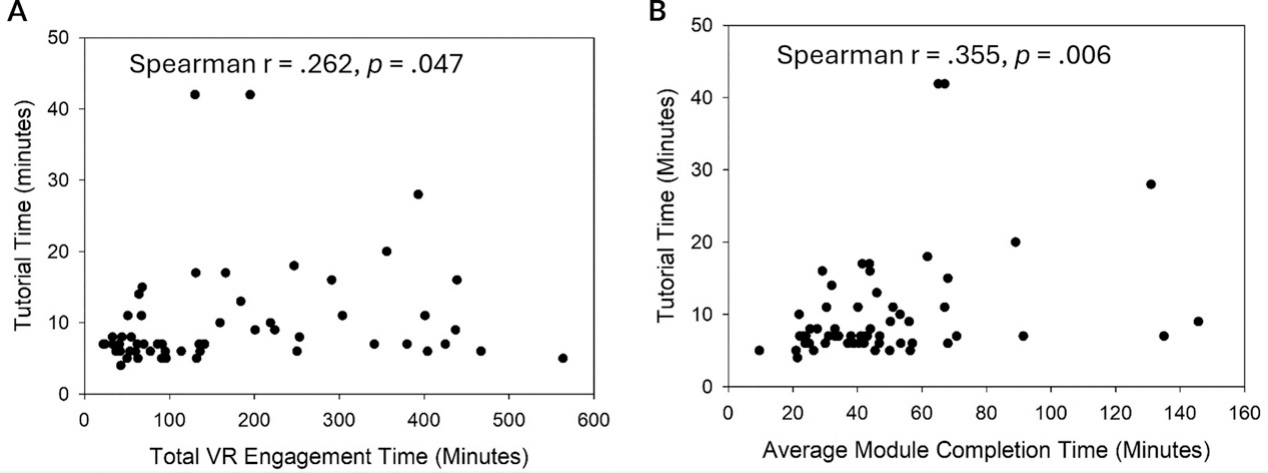
Scatterplots illustrate relationships between time spent completing the tutorial exercises for the Retreat VR program and each of total engagement time (Part **A**) and average Module completion time (Part **B**). VR, virtual reality.

In addition, we found that time spent completing the tutorial was positively correlated with both total engagement time (Spearman [rank-order] r = 0.262, *p* = 0.047) (Fig. 8A) and average module completion time (Spearman [rank-order] r = 0.355, *p* = 0.006) in the subsequent TST-VR modules (Fig. 8B).

We categorized circadian patterns of VR usage into four time segments (midnight to 6 AM, 6 AM to noon, noon to 6 PM, and 6 PM to midnight) and tallied the proportion of participants using the device at least once during each segment and calculated the total time spent within those segments. Descriptive values for each of the 4-Module and 10-Module program versions are shown in Table 3 and Table 4, respectively.

**Table 3.** 4-Module Time of Day Usage (*N* = 34)

	Midnight to 6 AM	6 AM to noon	Noon to 6 PM	6 PM to midnight
Unique users	5	12	34	12
Percentage of total users	14.71%	35.29%	100.00%	35.29%
Total time spent	219.9	710.1	2650.5	542.7
Total engagements	12	40	131	22

**Table 4.** 10-Module Time of Day Usage (*N* = 24)

	Midnight to 6 AM	6 AM to noon	Noon to 6 PM	6 PM to midnight
Unique Users	7	14	24	14
Percentage of Total Users	29.17%	58.33%	100.00%	58.33%
Total Time Spent	352.9	801.9	3482.9	1398.8
Total Engagements	15	37	168	70

**FIG. 9.**
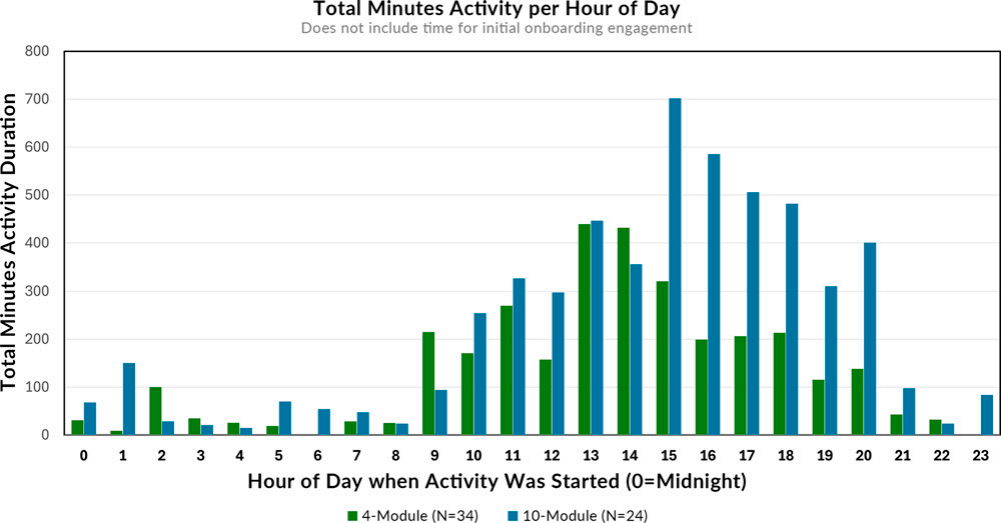
Column bars denote the incidence of time of onset of VR engagement (across all participants) along the X axis. The Y axis values denote the total time of VR engagement across all engagement instances in that hour block. Blue bars indicate values for the 10-Module version and green bars indicate 4-Module version. Because the initial module was at a prescribed time rather than one chosen by the user, the time data for the initial onboarding engagement was not included.

To provide a complementary description of “when” users chose to engage with the headset and a more granular view of temporal use patterns, engagement times were binned and summed for all users into the hour corresponding to when they started a given engagement (Fig. 9). Other than the initial onboarding and use of the headset, which was at a regular time each week, users were free to interact with the headset during any free time during the week or weekend.

## Discussion

The TST-VR program used in this study is novel in its design and approach of using VR to support SUD treatment. As opposed to cue exposure or craving induction approaches used in previous VR programs,^13^ TST-VR creates an environment, a virtual world to explore and engage in, where the patient can be repeatedly introduced to the same CBT-based principles being used in the patient’s conventional in-person or group therapy. In addition, the program has added supporting content such as instruction in several forms of meditation and mindfulness as well as additional modules covering core aspects from TST, such as acting in accordance with one’s moral compass to coping strategies for stress, anger, and anxiety.^3^ Beyond the primary instructional modules, the program offers several distraction-based experiences—for instance, a cross-country road trip, a balloon flight over snow-capped peaks, or a playful round of fetch with a virtual wolf. These short, self-contained scenes serve as deliberate attention-shifters: they potentially give participants a chance to regulate arousal, cultivate positive affect, and “reset” between instructional treatment blocks. The goal in increasing modules was to expand the topics covered as well as to increase engagement and user interest based on early feedback from users wanting more content. A consequence of including so many modules and approaches is that the material can become rather expansive, and it is critical that the user does not become disengaged with the program and discontinue use.

To our knowledge, there are no previous studies of engagement with this form of VR programming for SUD. At best, a close corollary may be analysis of engagement with smartphone applications for mental health. The use of mobile digital mental health applications has shown potential for providing timely support, easing the cost of mental health care, combating stigma, and enhancing therapeutic outcomes, but a common pitfall has been user engagement issues.^9^ Meta-analysis of smartphone applications have identified adherence rates as typically “sub-optimal,” with usage consistently declining over the course of a trial.^14,15^ While these smartphone applications are substantially different from a VR program both in design and usage, these analyses indicate the importance of identifying whether patient usage (adherence) would drop off as additional content was added to the program and more time was required of the patient to fully engage with this content.

The current findings strengthen the notion that a more expansive VR program can improve rather than hinder engagement among veterans undergoing residential SUD treatment. By comparing the original 4-Module version of TST-VR with a 10-Module variant, we observed that rather than being deterred by the prospect of a longer journey or assignment, participants exposed to the expanded program instead spent significantly more time in the VR headset and showed higher rates of module completion, as highlighted in Table 2 and Figure 6, providing a counterpoint to concerns that “more Modules” and a greater time commitment might increase dropout.^10^ These results are especially noteworthy considering the broader digital mental health literature, which has frequently documented problems with user attrition in mobile applications.^9,14^ In contrast, our data suggest that VR’s immersive nature may sustain motivation over a more extended course of treatment.

As mentioned above, in an exploratory analysis we decided to look further into temporal usage patterns, which may indicate potentially relevant insights. First, we examined whether the time spent in the tutorial was indicative of completion. The first thing a user does after a brief introduction to the program is to go through a tutorial to learn how to use the controls and navigate the environment. We were aware that some users had difficulty in this section. With a mean age of 50 for the 4-Module and 48 for the 10-Module versions (Table 1), we believe that many of these users may not have extensive experience with gaming devices. User-centered design changes improved the tutorial experience between the 4-Module and 10-Module versions but we envisioned a possibility wherein, for both versions, a higher tutorial time could be indicative of a technical challenge in mastering the interface, resulting in a user not completing all Modules. However, as seen in Figure 7, for the 10-Module group there was no difference in the average time spent in completing the tutorial between participants who did vs. did not complete all Modules. Conversely, in the 4-Module group, the median time spent in the tutorial were 15 min for those who went on to complete all Modules. The average time in the tutorial for those who completed it but did not finish all four Modules was only 7 min. Parenthetically, after excluding the two extreme outlier values in the 4-Module completers, this difference was statistically significant (*t* = 3.171, *p =* 0.019). Additionally, we tended to find evidence that minutes spent completing the tutorial was predictive of mean time in each subsequent module, and by extension, total minutes in the whole program (c.f. Fig. 8). While this was a small sample size, in the 4-Module version it is interesting that some users who may have struggled to get through the tutorial were more likely to finish all Modules. Unfortunately, we did not collect and record any immediate commentary from the participant or the staff member on perceptions of participant difficulty in mastering the virtual actions and navigation. This could be incorporated into future studies.

When we explored time-of-day usage (Tables 3 and 4; Fig. 9), a notable portion of participants, 29.1% in the 10-Module group and 14.7% in the 4-Module group, used TST-VR overnight between the hours of midnight and 6AM, highlighting the potential for VR-based interventions to fill critical gaps in care. In fact, the capacity for a VR program to be implemented within residential and outpatient therapy may thus complement standard therapy through delivering psychoeducation content and coping activities on demand. Future research might further examine whether overnight VR engagement correlates with reductions in nocturnal mood disturbances, cravings, or other significant clinical outcomes.

Interestingly, we observed increased total usage and a smaller median time in any particular Module for the 10-Module version relative to the 4-Module version (Figs. 3, 4). This aligns with the design intent when creating the 10-Module version to avoid overwhelming each individual Module while still expanding the breadth of content overall. It is notable that despite the differences between the 4 and 10 Module versions, the median user engagement time (the time from when a user puts on the headset until they take it off) shown in Figure 5 was almost identical, with a median of 20 min across both groups. Beyond just the expansion in overall content, users in the 10-Module program may have found the slightly shorter modules and other user-centered design changes more manageable, leading to repeated engagement and a higher rate of completion.

Despite these encouraging findings, limitations warrant mention. Our sample size was modest, and the study was not powered to assess clinical efficacy differences. It is hoped that this VR program will be studied in a larger RCT, which could also address these aspects as well as additional ones, such as whether individual differences, such as severity of SUD or comorbid PTSD, modify VR usage patterns.^1,2^ Moreover, participants were not formally randomized to the different programs. However, there were not likely any systematic differences in the overall SUD severity or other clinical markers between patients admitted to residential care before vs after the roll-out of the revised 10-Module TST-VR version. We note that the demographics of participants before and after the transition did not differ (Table 1).

Overall, the present data add to the growing body of evidence^3,6^ that immersive VR can meaningfully support SUD therapy by fostering a sense of immersion and ongoing engagement and providing a range of interactions from psychoeducational content to meditations to distraction-based content.

## Abbreviations Used

CBT: Cognitive Behavioral Therapy
HIPAA: Health Insurance Portability and Accountability Act
IQR: Interquartile range
NIST: National Institute of Standards and Technology
SD: Standard deviation
SEM: Standard error of the mean
SUD: Substance use disorder
TBI: Traumatic brain injury
TST: Transcending Self Therapy
TST-VR: Transcending Self Therapy Virtual Reality
VR: Virtual reality
VR-CORE: Virtual Reality Clinical Outcomes Research Experts (per Birckhead et al. framework)
